# Localized versus generalist phenotypes in a broadly distributed tropical mammal: how is intraspecific variation distributed across disparate environments?

**DOI:** 10.1186/1471-2148-13-160

**Published:** 2013-07-31

**Authors:** Diego F Alvarado-Serrano, Lucia Luna, L Lacey Knowles

**Affiliations:** 1 Department of Ecology & Evolutionary Biology, Museum of Zoology, University of Michigan, Ann Arbor, MI 48109-1079, USA

**Keywords:** Adaptation, *Akodon mollis*, Geometric morphometrics, Skull shape, Tropical mountains

## Abstract

**Background:**

The extent of phenotypic differentiation in response to local environmental conditions is a key component of species adaptation and persistence. Understanding the structuring of phenotypic diversity in response to local environmental pressures can provide important insights into species evolutionary dynamics and responses to environmental change. This work examines the influence of steep environmental gradients on intraspecific phenotypic variation and tests two hypotheses about how the tropical soft grass mouse, *Akodon mollis* (Cricetidae, Rodentia), contends with the disparate environmental conditions encompassed by its broad distribution. Specifically, we test if the species expresses a geographically unstructured, or generalist, phenotype throughout its range or if it shows geographically localized morphological differentiation across disparate environments.

**Results:**

Using geometric morphometric and ecomorphological analyses of skull shape variation we found that despite distinct environmental conditions, geographically structured morphological variation is limited, with the notable exception of a distinct morphological disjunction at the high-elevation forest-grassland transition in the southern portion of *A. mollis* distribution. Based on genetic analyses, geographic isolation alone does not explain this localized phenotype, given that similar levels of genetic differentiation were also observed among individuals inhabiting other ecosystems that are nonetheless not distinct morphologically.

**Conclusions:**

Instead of phenotypic specialization across environments in these tropical mountains, there was limited differentiation of skull shape and size across the broad range of *A. mollis*, with the exception of individuals from the puna, the highest-elevation ecosystem. The high morphological variance among individuals, together with a weak association with local environmental conditions, not only highlights the flexibility of *A. mollis*’ skull, but also highlights the need for further study to understand what maintains the observed morphological patterns. The work also indicates that mechanisms other than processes linked to local ecological specialization as a driver of diversification may contribute to the high diversity of this tropical region.

## Background

Phenotypic differentiation among closely related taxa along environmental gradients is well documented (e.g., [1-3]). For example, across species there is a negative correlation between body size and environmental temperature (Bergmann’s rule [4]) and an association in mammals between precipitation and high degree of hypsodonty in species inhabiting arid and grassy regions [5,6]. However, localized phenotypes may not necessarily be observed within a species, even if populations occur across steep environmental gradients and disparate ecosystems. Depending on the relative fitness trade-offs between alternative phenotypes, degree of population isolation, and the constancy and/or spatial heterogeneity of the environment [7-9], a geographically unstructured, generalist phenotype may be expressed throughout the species’ range that is capable of thriving in a wide variety of conditions.

Tropical montane ecosystems represent an ideal system to uncover the role of the environment in shaping intraspecific phenotypic variation as species experience substantial and often drastic different environmental conditions across montane ecosystems [10,11]. Temperature, air pressure, solar radiation, precipitation, area, cloud cover, soil quality, and productivity change considerably with elevation [12,13]. As a consequence, tropical mountains are characterized by a wide array of habitats with a highly patchy distribution given the high topographic heterogeneity of these mountains [11,13]. Such disparity in environmental conditions, together with the temporal environmental stability of tropical mountains is expected to promote specialization to local conditions [14,15], and hence, the expression of local phenotypes. Such a mechanism might promote speciation, generating the hyperdiversity observed in tropical mountains. However, other factors might counter the prevalence of local phenotypes. For example, depending on the degree of population connectivity across ecosystems and the extent of climate-induced distributional shifts associated with paleoclimatic conditions (e.g., [16]), a generalist phenotype strategy might be favored [8,17,18].

**Figure 1:**
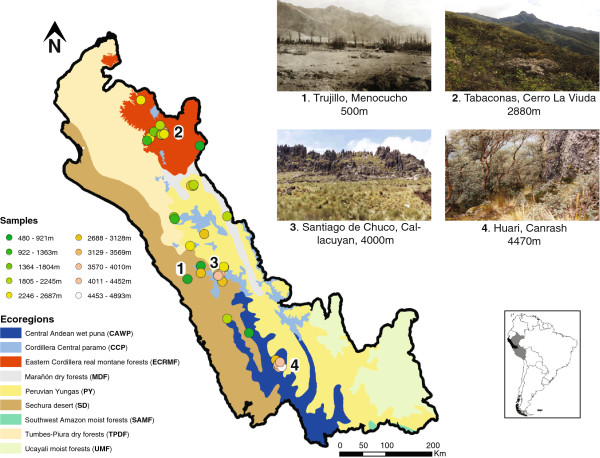
**Ecosystem map of northwestern Peru indicating the location of samples.** The inset map on the bottom right corner shows the position of Peru (in grey) and of the study region (in black) within South America. Pictures of the habitats of four of the localities (their location indicated by the numbers on the map, which follow the numbering of the pictures) are also included to show the abrupt transition between habitats along the altitudinal range of *A. mollis*. On the central-north and southeast Peruvian Andes the abrupt transition between forested and non-forested habitats occurs when the humid Andean cloud forest is replaced by an elfin forest [10,35], which itself is replaced at higher elevations by grasslands called páramos. These grasslands are characterized by high atmospheric humidity, high rainfall, and a continuous layer of short vegetation, dwarf shrubs, and wetlands [36-38]. On the central-south and southwest Peruvian Andes, this abrupt transition occurs between the xeric habitats characteristic of this region and a short forest dominated by quenoa trees (*Polylepis* spp.), which is then replaced by puna at higher elevations [39]. The puna ecosystem corresponds to steppes of isolated grasses and shrubs, low productivity, and an annual precipitation markedly seasonal and lower than that of the páramo [40-42]. Picture of Menocucho (locality 1 above), courtesy of The Field Museum, [CSZ36381].

Here we explicitly test for the existence of local phenotypes in the soft grass mouse, *Akodon mollis*, a tropical species with a widespread distribution that makes it ideal for examining how phenotypic variability is structured in taxa facing disparate environmental conditions that are relevant to exploring what evolutionary strategies might be used by taxa to cope with the steep environmental gradients of tropical mountains. This species ranges from approximately 500 to 5500 m in the Andes of Ecuador and northwestern Peru [19], across disparate habitats and steep ecological transitions such as the one between the tree-dominated Andean slopes and the grassland-dominated highlands (Figure 1). In contrast to many tropical Andean rodents that are acknowledged to be fairly specialized [20-22], the few ecological studies on this species suggest *A. mollis* is a widespread and an abundant generalist with relatively limited individual dispersal capabilities relative to other small terrestrial mammals [21,23,24]. Its diet is comprised of varying proportions of plant and insect material, like other species of the genus [21,23]. Yet, it remains unknown how *A. mollis* thrives in the broad range of environmental conditions encompassed by its distributional range, which is much broader than that of its close relatives [19,23]. Specifically, its distribution might reflect a wide tolerance owing to a geographically unstructured, generalist phenotype that is adapted to a broad range of conditions (hereafter, generalist phenotype strategy). Alternatively, the species may be characterized by local phenotypes, product of plasticity or local adaptation to local environmental conditions (hereafter, localized phenotype strategy). To investigate this issue, we focus on skull morphological variation because of its fundamental role in food processing and perceiving sensory information that allows vertebrates to interact with its environment [25], as evidenced by environmentally induced morphological changes in mice skulls [25,26]. For example, functional links between skull variation and environmental differences have been demonstrated by studies on the biomechanics of mastication under different diets [27,28] or differing selective regimes [29,30]. In particular, differentiation across disparate environments might be evident in areas of the skull that serve for muscle attachment (e.g., zygomatic arches [31]), influence bite force (e.g., rostrum [32]), or have been shown to covary with environmental gradients (e.g., the auditory bullae with aridity [27], or the palatal region with temperature [33]). Morphological differentiation across environments might also arise as an indirect effect of changes in performance under different conditions (e.g., increased energy available for investment in growth [34]).

Using a geometric morphometric approach and ecomorphological analyses, we test for two contrasting patterns of phenotypic variation. Specifically, we test for (i) a geographically unstructured, generalist phenotype strategy of limited morphological differentiation between populations across environments, or (ii) a localized phenotype strategy with morphological differentiation between populations from different environments, with a strong association between skull morphology and local environmental conditions.

## Results

A visual examination of the extent of size and shape variation in the skulls of *A. mollis* individuals across environments indicates high intrapopulation variability, where size and shape variation was measured by the log_e_-transformed centroid size [43] and by all non-zero-variance principal components of a PCA on the covariance matrix of either the size-corrected (i.e., controlling for allometric effects; see Methods), or uncorrected, symmetric component of the landmark configuration [44], respectively. Moreover, limited morphological differentiation among populations and ecosystems is also apparent, with the exception of those from the highest ecosystem of the Central Andes – the wet puna (hereafter, referred to as puna; Figure 1); note that ecosystems correspond to biophysically defined regions ([45]; see below for additional discussion between ecosystems and environmental conditions).

**Figure 2:**
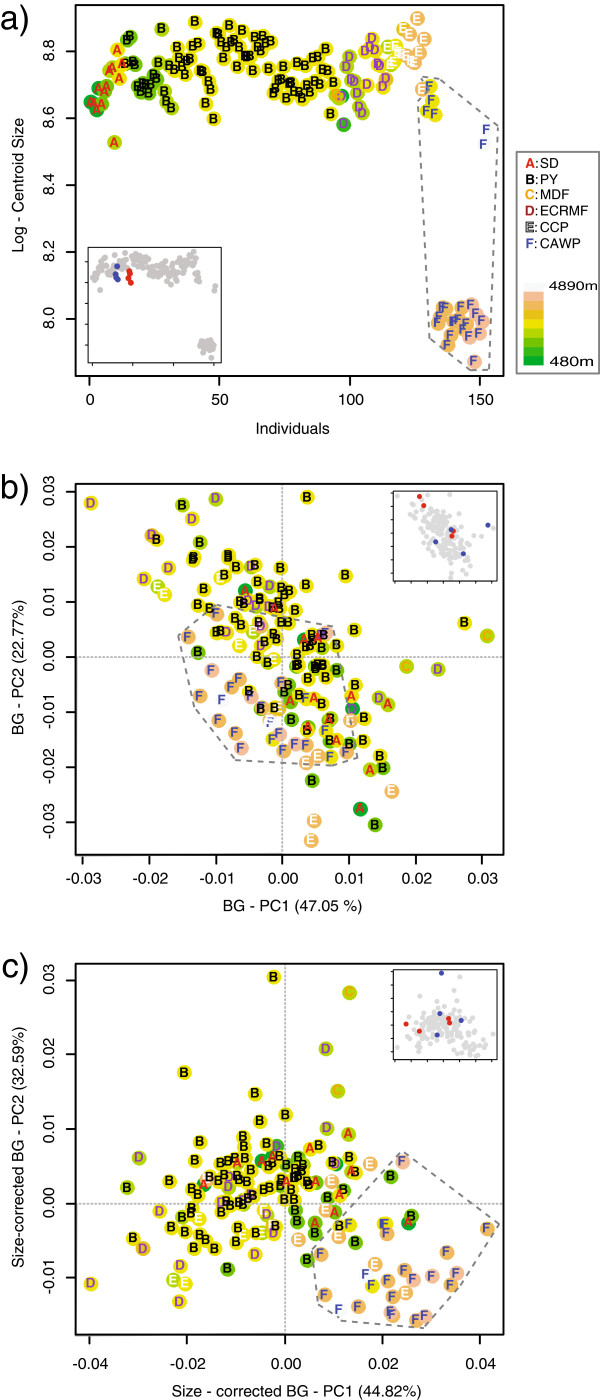
**Skull size and shape variability in *A*. *mollis.*** Scatterplots of all sampled individuals according to their centroid size **(a)** and their scores on the first two between-groups principal components (BG-PCA) of uncorrected **(b)** and size-corrected shape **(c)** (see Methods for details). Individuals from the highest ecosystem (i.e., Central Andean wet puna) are surrounded by a minimum convex hull to facilitate comparison between scatterplots. In addition, in order to illustrate the amount of intrapopulation variance, the position of individuals from two arbitrarily selected populations are highlighted in blue and red (populations 9 and 14, respectively; see Appendix) as an example in an inset in each plot. Symbols are based on two proxies of environmental variation, the elevation and ecosystem (abbreviations as in Figure 1) in which each specimen was sampled.

Individuals from the puna are distinguished from all those from other ecosystems by their smaller average size (Figure 2a), which is significant based on an analysis of variance (ANOVA) with ecosystem as a fixed factor (F_5_ = 87.82, p-value < 0.01 and < 0.01, based on a parametric and resampling approach, respectively; see also Additional file 1). A between group-PCA (BG-PCA [46]), in which shape data averaged by ecosystem were used to construct the principal axes onto which individuals were then projected, was also conducted. This analysis shows similar, but more limited, patterns of morphological differentiation of puna individuals, as can be seen in a plot of the first and second BG-PCA axes for the size-corrected and uncorrected shape data (Figure 2b, c), which together account for around 70% of the between-group variance in both analyses (surprisingly this pattern of differentiation is more evident in the size-corrected data than in the uncorrected data; see results and discussion below). It is important to note that similar levels of morphological differentiation of the high-elevation puna individuals was observed when the BG-PCA axes were constructed based on elevation groups, as opposed to ecosystems (results not shown), which suggests the results are not an artifact of the *a priori* groupings used.

**Figure 3:**
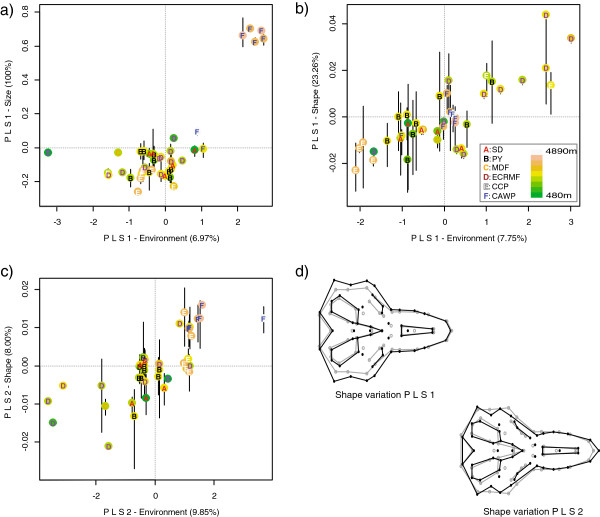
**Morphological-environmental association recovered in the PLS analyses.** Scatterplots of the single set of PLS axes for the analysis on size **(a)**, and the first **(b)** and second **(c)** set of PLS axes for the analysis on uncorrected shape (results for the size-corrected shape data were similar; not shown). Although PLS analyses were run on population-averaged data, the extent of local intra-population variability is depicted as vertical lines below and above each population score, with the line lengths determined by the minimum and maximum individual scores obtained for each population when all individuals are projected onto each morphological PLS component. In **(d)** the morphological changes associated with the PLS axes shown in **(b)** and **(c)** are summarized by comparing the skull regions associated with each PLS (in black) with the overall mean shape of the entire sample (in grey). Scatterplot symbols follow Figure 2.

To statistically explore the degree of morphological and environmental covariation PLS analyses were conducted (note the BG-PCA, used above, is a graphical technique; see Methods for details). Population averages were used in these and the SEVM analyses (see below) because of unequal sampling across ecosystems (Table 1), which reflects natural differences in the abundance of *A. mollis*[23], but may nonetheless compromise statistical analyses based on individuals (no formal disparity analysis was feasible given the sample size differences across populations). The results of these analyses show that although size and shape variation significantly covaries with environment (RV coefficient = 0.15, 0.31, and 0.30 with p-value = 0.02, < 0.01, and < 0.01 for size, uncorrected shape, and size-corrected shape, respectively), the amount of morphological and environmental variance contained within the PLS axes is limited. In fact, less than 7% of the environmental variance was explained in the PLS analysis of size (Figure 3a), which is the primary phenotypic feature that distinguishes the individuals from the puna from those of other ecosystems (Figure 2). Moreover, in none of the analyses of shape data did the first two PLS axes explain more than 36% or 18% of the morphological and environmental variance, respectively (Figure 3b, c).

**Table 1:** Sample size of individuals available for the analyses

**Ecosystem**	**No**. **of localities**	**No**. **of individuals**	**Mean no**. **of individuals per locality**	**No**. **individuals by locality (♀/♂)**
CAWP	7	24	3 ±2	[1]: 0/2; [2]: 0/5; [3]: 2/2; [4]: 1/1; [5]: 4/2; [6]: 0/1; [7]: 2/2
CCP	7	15	2 ±1	[18]: 1/3; [19]: 1/1; [28]: 0/1; [29]: 0/2; [30]: 0/2; [31]: 0/1; [32]: 1/2
ECRMF	10	18	2 ±2	[15]: 1/0; [16]: 2/4; [17]: 0/1; [34]: 0/1; [35]: 1/0; [36]: 0/1; [37]: 1/0; [38]: 0/1; [39]: 2/2; [40]: 0/1
MDF	1	2	2 -	[10]: 1/1
PY	10	81	8 ±13	[9]: 1/3; [11]: 1/1; [12]: 1/1; [13]: 21/23; [14]: 2/2; [20]: 1/2; [21]: 2/0 [22]: 6/2; [25]: 3/6; [26]: 1/2
SD	5	13	3 ±1	[8]: 1/2; [23]: 2/1; [24]: 1/1 [27]: 1/0 [33]: 0/4

The number of males and females per population, the number of localities comprised by each ecosystem (abbreviations as in Figure 1), and the mean and standard deviation of the number of individuals per locality are provided. Localities with individuals available for genetic analyses are indicated in bold; locality number, between square brackets, follows appendix, follows Appendix.

A scatterplot of the first set of PLS components of the size analysis shows that the association of skull size with environment is mainly driven by the smaller size of the majority of puna individuals (the single PLS of morphology in this analysis is negatively associated with skull size) (Figure 3a). In contrast, scatterplots of the shape data for the first 2 sets of components show a linear pattern of covariation between morphological and environmental variation (Figure 3b, c). In this latter analyses, the first morphological PLS axis, which is significantly correlated with latitude (Pearson’s r = 0.42, p-value = 0.007), mostly reflects differences in the volume of the braincase (Figure 3d) and presents no obvious association with ecosystem. The second morphological PLS axis separates populations according to elevation (Pearson’s r = −0.55, p-value < 0.001) by differences associated with multiple regions of the skull that affect both the breadth of the braincase and the length of the rostrum (Figure 3d). The morphological distinctiveness of puna populations does not correspond to latitudinal environmental variation (see below for a discussion of the contribution of spatial and genetic isolation to the distinctiveness of the puna phenotype), but instead to differentiation of mid- and high-elevation habitats (Figure 3c). It is important to note that the fact that puna individuals appear as partially distinct only in the second set of PLS axes is not necessarily unusual because PLS axes are not expected to capture the main axis of morphological differentiation given that they do not maximize the within block variance [47,48].

**Figure 4:**
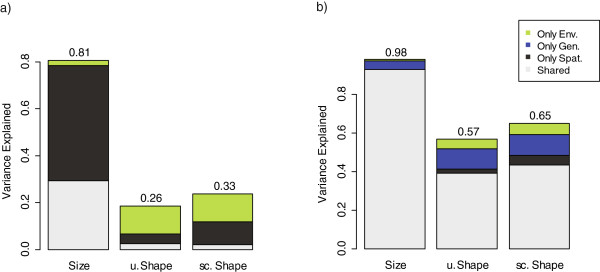
**Relative explanatory power of environmental variation in SEVM regressions.** Result of spatial regressions of size and uncorrected (u.) and size-corrected (sc.) shape variation on environmental variables **(a)** or on environmental and genetic variables **(b)**. The proportion of variance explained exclusively by each set of predictors as well as the shared explained variance (i.e., combined effect of spatial and environmental in **(a)**, combined effect of spatial, genetic, and environmental effects in **(b)**) is presented. The total variance explained is given on top of each bar. Note that in the shape analyses, the data depicted correspond to the overall variance explained (i.e., weighted sum of the variance explained in the regressions for each shape PC retained, see Methods, with weights determined by the proportion of variance explained by each shape PC).

**Figure 5:**
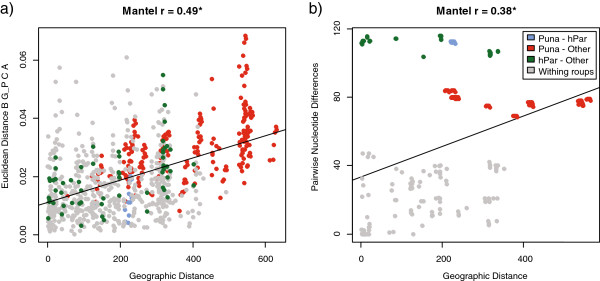
**Morphological and genetic spatial isolation.** Scatterplot of pairwise morphological **(a)** and genetic **(b)** distance against pairwise geographic distance between populations. Morphological distance was calculated as the Euclidean distance between population-averaged scores on the first 2 BG-PCA axes of the size-corrected shape data (see Figure 2c), whereas genetic distances was calculated as population pairwise nucleotide differences in the cytochrome-b sequences. Points are colored according to which ecosystem the populations being compared pertain (i.e., puna, high-elevation páramo, or other population). The correlation coefficient and significance of both morphological and genetic trends are presented above each scatterplot. Note that the greatest genetic distances do not correspond to comparisons involving puna populations, but to those involving high páramo populations (named hPar in legend; see Additional file 2).

The robustness of the morphology-environment association to spatial autocorrelation of environments was verified by running Spatial EigenVector Mapping regressions (SEVM; [49,50]). In these analyses, size variation is not significantly correlated with environmental variation after controlling for spatial autocorrelation (Figure 4a), whereas overall shape variation (uncorrected and size-corrected) is significantly correlated with the environment, but only explains about 10% of the total shape variance (estimated as the weighted sum of the variance explained by the regressions of each principal component, see Methods; Figure 4a). This relationship is robust after accounting for genetic relatedness (Figure 4b). Yet, after controlling for genetic contributions, the environment explains less than 6% of the total shape variance, with genetic relatedness explaining approximately 4% and 12% of the size and shape variance, respectively. The reduction in the environmental effect after accounting for spatial and genetic factors is in line with a significant effect of spatial isolation (Mantel test *p*-value < 0.001) on patterns of morphological and genetic differentiation (Figure 5), affirming the other analyses (discussed above) that suggest the lack of a strong effect of the environment on morphology.

## Discussion

The analyses revealed limited support for local phenotypic structuring of the soft grass mouse, *A. mollis,* given the lack of consistent morphological differentiation of the skull across environments (Figures 2 and 3). Although a significant covariation trend between environmental and size and shape variation is recovered (Figure 4), it explains limited amount of the phenotypic variance, and is on the same order as the exclusive contribution of genetic and spatial isolation (Figure 4b). Moreover, the association between the environment and size and shape variation would no doubt be (at least in part) weakened if the extensive amount of local morphological variability was considered (Figure 3; as noted in the results and methods, population means instead of individuals were analyzed to avoid the confounding problems of population sample sizes). These findings suggest that *A. mollis*’ skull morphological variation related to local environmental conditions have a significant, but limited, effect on overall skull shape and size. The primary exception to what appears to be a geographically unstructured generalist phenotype is the high-elevation puna Andean ecosystem in the southern portion of *A. mollis* distribution. This group of individuals is consistently morphologically distinct from individuals from the other ecosystems across the majority of analyses – it even differs from populations from similar elevations but different ecosystem (i.e., páramo; Figure 2). Taken together, these results suggest that the skull of this species fits neither a strict generalist phenotype strategy (i.e., a geographically unstructured phenotype throughout its range) nor a strict localized phenotype strategy (i.e., different local phenotypes).

### Limited morphological specialization in the skull?

The limited amount of geographically structured morphological differentiation observed throughout most of the broad environmental gradients *A. mollis* experience is noteworthy in light of previous studies that have shown that skulls commonly vary within species in response to environmental conditions [51,52]. Not only have previous studies uncovered moderate to considerable levels of size and shape differences in vertebrate’s skulls due to diet, latitude, temperature, elevation, precipitation, seasonality, and/or productivity, or combinations of these variables [53-55], but mice in particular have been shown to experience significant and rapid morphological changes in response to changes in temperature, precipitation, and human population density over the last 100 years [56].

The weak environment-morphology association is not attributable to a general lack of morphological or genetic variation (Figure 5, see also Additional file 2). To the contrary, the species is characterized by substantial morphological variability (even if we exclude the differences between puna and the other populations, Figures 2 and 3; also see [23,57,58]) and genetic variation, with relatively large average pairwise sequence divergence between individuals (5.6 ± 4.9% for all data, and 4.83 ± 5.41% when puna individuals are excluded, Additional file 2). In contrast to other mice with broad geographic ranges that do show localized morphological differentiation (e.g., [25,33]), the observed variation among individuals within populations suggests that the absence of local specialization may result from a relaxed association in *A. mollis* between skull morphology and ecological performance (cf. [59]) (i.e., relaxed selection), especially considering that the variation is present irrespective of whether individuals experience similar biotic and abiotic conditions (i.e., occur at the same locality). Under this hypothesis, environmental canalization of skull morphology would be relaxed due to weakened effective selection against alternative phenotypes, possibly because of small effective population size or by recent or frequent environmental changes [60], for instance, due to human-induced landscape changes or natural climatic events. Alternatively, the variability might reflect a general ecological plasticity in the species [21,23], which might be favored to increase the species’ ecological breadth [17]. In this case, the observed morphological variation among coexisting individuals could reflect a strategy to reduce intraspecific competition under high local abundances (typical of *A. mollis*) and maintain high adaptability potential [61,62], as suggested for some other generalist vertebrate species [63,64], including some species of mice [65]. Under this latter scenario, the microhabitat experienced by an individual throughout development may be more important in determining skull variation in ecological-plastic species than macro-environmental conditions (i.e., the differences in habitat that is captured by the coarse-grained variables used here). Individual variation under this scenario could arise for instance from variation in individuals’ diet, as have been observed in feeding trials in deer mice [26]. Testing of these alternatives (and at this point, speculative scenarios) requires detailed eco-morphological studies of multiple cohorts through time, and hence, goes beyond this contribution. Nevertheless, future studies on this topic might significantly add to our understanding of the role that environment plays in morphological evolution and ecological specialization.

It remains to be seen if similar patterns of morphological variation are observed in other tropical generalist species. It is possible that *A. mollis* may be exceptional among tropical species, especially considering the narrow elevation ranges and high species turnover typical of tropical mountains [11,13,66]. However, our results raise questions about the general expectation that locally adapted populations will necessarily predominate in the tropics [14,15]. Moreover, our results pose the question of whether the general lack of strong geographically structured morphological variation observed in *A. mollis* (with the exceptions and possible caveats discussed above) may be in part responsible for its exceptional environmental tolerance and broad geographic distribution (cf. [17]), and highlight the contingency of eco-morphological patterns on the natural history and geographic setting of the species involved.

### Uniqueness of puna individuals

Although *A. mollis* generally shows limited evidence for geographically structured morphological phenotypes (Figures 2 and 3), there is of course the exception of the morphological distinction between individuals from the puna ecosystem and the rest of populations. These distinct individuals are noticeably smaller and partially distinguishable both in uncorrected and size-corrected shape components of variation (most evidently so in the size-corrected dataset; Figure 2) – note that such distinction seems unlikely to be a sampling artifact given the magnitude of the size differences (Figure 2a). However, why such a morphological break is observed here, but not between other ecological transitions over environmental gradients that are just as extreme (Additional file 1) remains unexplained (see below for further discussion).

It is possible that this morphological distinctiveness may result from the particular type of ecological conditions associated with the puna ecosystem, and specifically, the transition from the treeline to the grassland, which has an important effect on microhabitat conditions that are not necessarily captured by the coarse-grained variables used here. The progressive disappearance of tree coverage at high elevations diminishes the buffering effect of vegetation on microclimatic conditions [67,68]. Consequently, the physical conditions organisms have to cope with at high tropical elevations become harsher as average temperatures, atmospheric pressure, atmospheric concentration of oxygen, and water vapor pressure decrease, and daily temperature fluctuations and physiological aridity increase [40,67,69]. Although similar challenges are experienced by individuals inhabiting high-elevation páramo environments, the more arid conditions of the puna ecosystem ([36,41]) and the average higher and more southern location of puna populations, may exacerbate the cold-desertification and hypoxia effects of high elevation environments. In line with this hypothesis, the first and second environmental PLS axes in the size and shape analyses, respectively, along which puna populations are the most different (Figure 3c), are strongly associated with the first environmental principal component, which in turn is highly correlated with precipitation variables (Table 2; Additional file 3).

**Table 2:** Summary of PLS axes for size and shape analyses

	**Size**	**Uncorrected shape**		**Size**-**corrected shape**	
**Environmental PCs**	**PLS1**	**PLS1**	**PLS2**	**PLS1**	**PLS2**
PC1 (mean temperature)	−0.61	0.27	−0.68	0.58	−0.58
PC2 (annual precipitation)	−0.16	0.37	0.16	0.38	0.22
PC3 (precipitation maximum)	−0.39	−0.44	−0.36	−0.22	−0.52
PC4 (vegetation coverage)	−0.20	−0.12	0.05	−0.03	−0.09
PC5 (productivity + vegetation)	0.12	−0.26	−0.10	−0.27	−0.21
PC6 (seasonality + vegetation)	−0.10	−0.63	−0.08	−0.53	−0.27
PC7 (isothermality)	0.07	−0.25	0.40	−0.31	0.24
PC8 (temperature seasonality)	−0.47	−0.17	0.23	0.01	−0.09
PC9 (summer precipitation)	−0.23	0.08	0.35	0.10	0.33
PC10 (precipitation seasonality)	0.33	0.07	0.16	−0.08	0.23

Loadings of the 10 environmental principal components (PC) on the first set of PLS axes for the analyses on size, uncorrected, and size-corrected shape. The main environmental information summarized by each component is presented in parenthesis (note, however, that this is an oversimplification; see Additional file 3).

All these environmental changes, which are also associated with a significant turnover of resources that likely impact the diet of *A. mollis*, may contribute to different biomechanical pressures on puna individuals cf. [70,71]. This possibility seems feasible when considering that a previous study of the diet of other species of *Akodon* in southwestern Peru revealed a significant relationship between the proportion of insects in the diet and elevation [72]. Furthermore, similar changes in diet and associated morphological traits with elevation have also been observed in other cricetid mice [69,73]. Thus, it is certainly plausible (although purely speculative at this point) that such interactions between direct physiological constraints imposed by the challenging environmental conditions of tropical highlands and indirect pressures imposed by dietary constraints might be partially responsible for the differences in skull morphology observed in the high-elevation populations of *A. mollis*, as in cricetid mice [25,26].

Alternatively, it is possible that the morphological distinctiveness of the puna individuals is caused by some degree of genetic isolation of these highland populations. As seen in other systems, where steep ecological transitions are present, genetic isolation and strong phenotypic differences can arise and be maintained even between populations in close proximity [74-76]. Our preliminary exploration of levels of population genetic differentiation seems to partially support this possibility. However, unexplained patterns of genetic variation suggest that this alone cannot explain the morphological distinctiveness of puna populations. For example, the average cytochrome-b pairwise nucleotide differences between the populations for which we have genetic data showed that both puna and some high-elevation páramo populations differed markedly from the rest of populations (Additional 2). Moreover, these high-elevation páramo populations are genetically distinct from populations that are geographically proximate to them (and more so than the puna populations are from the other populations) (Figure 5b). Yet, unlike the puna individuals, there is only limited morphological differentiation between these high-elevation páramo populations and all other populations. These observations make it unlikely that the morphological distinction of puna individuals is exclusively driven by their degree of genetic isolation. Still, this question deserves further exploration given the claim that *A. mollis* may be a species complex has been made, albeit based on limited sampling [24].

Based on consideration of other genetically divergent populations, and in particular some populations from the páramo, it seems that the observed phenotypic distinctiveness of the puna individuals is not simply a function of the abiotic and biotic conditions unique to high elevation environments and their environmental isolation from lower environments [68], which could prevent the dilution effect of gene flow from lower populations [77]. One possibility is that divergence of the puna populations reflects the interplay of stochastic and deterministic factors. For example, recent work demonstrates how species divergence may reflect an interaction of past demographic conditions (including the stochastic effects associated with changes in population size) that accompanies climate-induced distributional shifts and the ecological attributes of species (including whether the taxon tracks shifts in their habitat and whether they are spatially restricted owing to particular habitat affinities) [16]. Hypothetically, for instance, if puna populations have been unable to migrate out of the puna due to its environmental isolation, it is plausible that they have been forced into a more specialized morphology due to the marked cycles of aridification this ecosystem experienced during the Pleistocene [78]. Additional studies that specifically address the origin and phylogenetic relationships of these genetically distinct populations are required before a more definitive conclusion about the validity of this hypothesis is reached.

## Conclusions

Even though high levels of ecological specialization has been proposed as a principal mechanism for generating tropical diversity [14,15], the ecomorphological analyses suggest that morphological specialization in the skull of *A. mollis* is limited, except for the localized phenotype of individuals from the puna. This finding suggests that *A. mollis*’ skull does not fit either a strict generalist or localized phenotype strategy, but a mix of the two, with limited geographically structured morphological variation thorough most of its broad geographic range. Together these results offer insights into the processes that generate and maintain diversity in the tropical Andes, a region that has been previously proposed to be an important center of differentiation and diversification [79-81]. Aside from the morphological disjunction identified between puna populations and the rest of populations, the lack of consistent and strong morphological differentiation across environments, and wide geographic range of *A. mollis* suggest that additional mechanisms of diversification in tropical systems may deserve more attention than they have received. The geographic structure of phenotypic variation in the skull of *A. mollis* may be exceptional considering that other morphological structures of this species have not been studied in *A. mollis *and the wide ecological tolerance of this mouse compared to other tropical species. Nevertheless, with these caveats in mind (as well as those regarding the interpretation of results given the small sample sizes of some populations) our results also have important implications for understanding biological resilience to environmental change in tropical mountains. Specifically, even though tropical mountain habitats are predicted to be disproportionally susceptible to ongoing environmental changes [82,83], the apparent adaptability of *A. mollis* suggest that it may be able to accommodate a broad range of conditions, and hence, be more resilient. Future studies that assess the generality of these findings and explore its mechanistic basis are expected to further advance our understanding of morphological evolution in one of the most diverse ecosystems on Earth.

## Methods

### Data acquisition

Morphological variation was assessed in the complete skulls of 153 *A. mollis* individuals sampled across its entire geographic range in Peru (Figure 1; Table 1). Specimens were obtained from the Museo de Historia Natural “Javier Prado”, Universidad Nacional Mayor de San Marcos (UNMSM) in Lima, Peru and the Field Museum of Natural History in Chicago (FMNH), US, and collected by L.L. in northern Peru following the American Society of Mammalogists’ guidelines [84] and University approved procedures for the manipulation of mammals (see Appendix). Given the taxonomic complexity of the genus [58,85,86], and the poorly defined diagnosis available for *A. mollis*, we based our taxonomic identification of specimens on a combination of external and internal morphological characters (e.g., a robust braincase, short rostrum, zygomatic plate broad and not inclined [24,57,87,88]). Only adult specimens were used to minimize the effects of ontogenetic differences [24,89]. Adults were defined as individuals with all three molars completely erupted and with the posteroloph in the third molar eroded (which corresponds to ages 3 to 5 [90]). Sex and locality information was recorded for all 153 specimens from skin tags and collectors field notes. When available, the locality’s latitude and longitude was also recorded. Otherwise, specimens were georeferenced based on the collector’s notes following a point-radius method [91]. We assessed the confidence of our georeferencing by contrasting the elevation given in the field and museum notes with one we obtained from a Digital Elevation Model (DEM) from the Shuttle Radar Topography Mission (SRTM [92]) based on the coordinates estimated.

For each locality we compiled information from 21 ecogeographical variables (Table 2) that have been shown to be of biological relevance in a wide variety of vertebrates [93,94]. These variables include summaries of temperature and precipitation patterns [95], including both mean, maximum and minimum values, as well as vegetation cover [96] and productivity [97]. To minimize the problem of collinearity among these environmental variables we run a PCA on these data and used the resultant orthogonal components in our analyses (Table 2). Because of the resolution of the ecogeographical data (climatic data: 1 km^2^, vegetation cover and productivity: 0.25 km^2^, and elevation: 0.02 km^2^), localities in close proximity (i.e., less than 1 km apart) were treated as a single locality, resulting in 40 unique localities (see Appendix). All geographic analyses were performed in ArcGIS v.9.3 [98].

In addition, for 95 individuals for which we were able to obtain tissues (these individuals were collected in 22 of the 40 populations) we extracted DNA using a Qiagen kit and amplified a 1123 bp-region of the mitochondrial cytochrome b gene following the procedures outlined by Smith and Patton [58]. After sequencing, these fragments were cleaned in Sequencher v4.6 [99] and aligned in MacClade v4 [100]. A haplotype frequency matrix was extracted from the aligned sequences and used in a PCA on the covariance matrix (for details see [101]). Individuals’ scores on the first three principal components, which together accounted for 71.66% of the genetic variance, were averaged by population and used to control for the effect of genetic distance in the spatial regressions (see below). In addition, and in light of the eco-morphological results, we performed an Isolation By Distance analysis (IBD, [102]) using Mantel tests [103] based on 10000 permutations to assess the spatial structure of the morphological and genetic data.

### Morphological analyses

**Figure 6:**
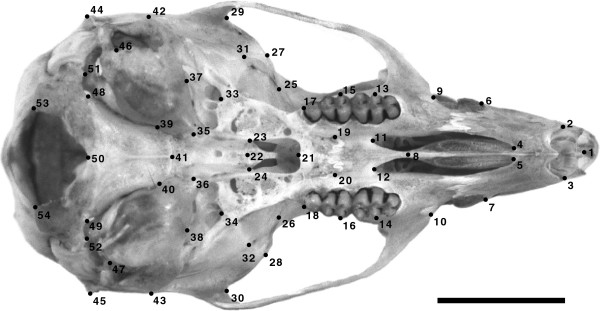
**Landmarks used to characterize the ventral skull of *A. mollis.*** Landmark description is provided in Additional file 4 Scale bar = 5 mm.

Ventral images of the 153 skulls were taken using a digital photographic camera Nikon D80 under standard conditions. The focus of the image was always set at the posterior margin of the palate, while keeping the molars of both tooth rows at the same plane. The image of each skull was used to digitized 54 two-dimensional, X, Y landmarks (Figure 6; Additional file 4) using tpsDig, version 2.16 [104]. The 54 landmarks, which follow the standards proposed by Zelditch *et al*. [48], were selected after an analysis of digitizing and photographic errors based on five randomly selected specimens that were photographed twice and digitized four times in random order by the same person. Landmarks for which raw coordinates showed significant differences among replicates, as identified using univariate analyses of variance (ANOVA) under a block design with individuals as fixed factor, were not used in the study.

The digitized landmarks were standardized using a full Procrustes superimposition followed by an orthogonal projection into the tangent space [105,106] to remove the effects of position, size, and orientation [48,107]. After superimposition, we searched for outliers in the dataset (i.e., individuals with landmarks that strongly deviate from the mean shape); outlier individuals were redigitized to assure they were not the product of digitizing error. We then used the vetted superimposed data to estimate size and shape skull components. Skull size was quantified as the centroid size of the superimposed landmark configuration, calculated as the square root of the sum of all squared distances between each landmark and the configuration centroid [43]. To minimize departure from normality, the log_e_-transformed centroid size was used in all analyses. Skull shape was quantified using the symmetric component of the morphological variation to avoid redundancy and to control for assymmetric differences in the skull [44]. Specifically, skull shape was quantified by all non-zero variance components of a Principal Component Analysis (PCA) on the covariance matrix of the symmetric coordinates.

Sexual dimorphism on size was assessed using a standard sex by locality ANOVA on the log_e_-transformed centroid size with locality as a block factor, whereas sexual dimorphism in shape was assessed by testing for differences in the scores on the shape principal components obtained using a sex by locality MANOVA. Both the ANOVA and MANOVA were run in R v2.15.1 [108]. Because no sexual dimorphism was detected on size (F_1_ = 0.67, *p* = 0.42; *p*-value interaction term = 0.89) or shape (Wilks’ λ = 0.38, F_54/38_ = 1.15, *p* = 0.33; *p*-value interaction term = 0.09), all specimens were pooled for further analyses.

Allometric effects were assessed by running independent regressions of the symmetric coordinates onto log_e_ centroid size for different sets of individuals grouped according to the ecosystem in which they were collected (ecosystems definitions followed [45]; Additional file 5). We verified the presence of similar allometric trajectories in all groups (i.e., similar regression slopes) in tpsRegr [109]. Although differences in regression intercepts were recovered (Wilks’ λ < 0.01, F_432/154.8_ = 2.72, *p* < 0.01), no significant slope differences were found (Wilks’ λ <0.01, F_432/138.8_ = 0.98, *p* = 0.56). Because the redundancy of symmetric data might compromise the significance obtained, we verified the robustness of the results by running a Multivariate Analysis of Covariance (MANCOVA) on the non-zero variance principal components of shape using the log_e_-centroid size as a covariate and ecosystems as groups in R v2.15.1 [108]. Since the results were consistent (i.e., neither the main effect of sex, nor the interaction term in the MANCOVA were significant [43], Wilks’ λ = 0.08, F_260/453.83_ = 0.98, *p* = 0.15), we used the residuals of a regression of the symmetric coordinates onto log_e_-transformed sized, pooling all individuals together, as a size-corrected shape component. We then run a PCA on the covariance matrix of these residuals and kept all non-zero variance components as our size-corrected shape component for all analyses.

### Variability assessment

The extent of among individual variability in skull size was visually assessed by plotting the log_e_-centroid size of specimens ordered according to the elevation at which they were collected; note that elevation is a well-suited proxy for environmental differences in tropical regions where elevation represents the most evident axis of climatic and ecological variation [68]; see Table 1). Uncorrected and size-corrected skull shape variation was visually assessed using between-groups PCA (BG-PCA, [46]), which is a modification of the most-commonly-used PCA that takes into account *a priori* individual grouping by identifying PCs from the variance-covariance matrix of group mean shapes and then projecting all individuals into these components [110]. We chose this approach as it represents a compromise between a PCA, which is a suboptimal analyses for revealing group differentiation, and a Canonical Variate Analysis or Discriminant Analysis, which is prone to over-identify group differences [110]. Specifically, we constructed BG-PCA axes using the ecosystem in which individuals were collected.

### Ecogeographic analysis

Given the highly unequal sample sizes across populations (Table 1), which reflects natural differences in the abundance of *A. mollis*[23], we averaged all size and shape data by population for the following analyses. In the case of shape data, we chose to average the scores of the uncorrected and size-corrected shape PCAs run on individuals (see details above) instead of averaging the landmark coordinates by population first and then running PCAs on the averaged data to avoid maximizing differences between populations. All analyses hereafter were performed on the population-averaged size and shape components. To explore the association between environmental conditions and morphological variation we performed 3 independent two-block Partial Least Squares analyses (PLS). We chose this method because it is robust to the underlying structure of the data as it does not rely on a particular variable model [48]. The first two PLS analyses were based on the averaged-by-population PC scores of all non-zero-variance PCs from either uncorrected or size-corrected data (as one block) and the first 10 principal components of the standardized environmental data (as another block, see Data acquisition section), which together accounted for 99.91% of the environmental variance. We also performed a third PLS analysis between averaged log_e_ centroid size and the same 10 principal components of the environmental data. In all three cases we summarized the overall strength of the association using the RV coefficient, which is a multivariate statistic analog to the squared correlation and ranges from 0 (uncorrelated) to 1 (completely correlated) [106,111,112].

In addition, to investigate the degree to which environmental conditions explain differences in skull size and shape after accounting for the effect of spatial autocorrelation (because skull similarity decreased as a function of distance between populations – evidenced by Moran’s I correlogram for size and Mantel’s correlogram for shape; results not shown), we run Spatial EigenVector Mapping regressions (SEVM; [49,50]). We chose SEVM because it has been shown to minimize spatial autocorrelation more efficiently than other techniques [50]. Using this spatially-explicit technique we performed a regression of the log_e_-transformed centroid size onto the first three principal components of the environmental data, which together accounted for 87.66% of the environmental variance, in SAM [113] –we chose 3 instead of 10 environmental components to minimize the risk of overfitting given our sample size. We then repeated these analyses on the population-averaged scores of all the non-zero-variance principal components of the uncorrected and size-corrected data. We combined the results of these regressions, separately for the uncorrected and size-corrected data, by adding the amount of variance explained by the SEVM regression on each component, weighted by the corresponding proportion of variance explained by each principal component. Finally, for the subset of our sample of populations for which we had genetic data (22 of 40 populations), we ran additional SEVM regressions that include the same first three principal components of the environmental data and additionally the first three principal components of the genetic data as predictors (see Data Acquisition section). For all SEVM analyses we used the automatic selection procedure of SAM to estimate the truncation distance and the spatial filters to use. We verified the effectiveness of these procedures by analyzing the degree of spatial autocorrelation of the regressions’ residuals using Moran’s I correlograms. Unless otherwise noted, all analyses were run in MorphoJ [106].

## Appendix – specimens used in the analysis

The 153 specimens used in the analyses are listed below. Geographical coordinates and elevations were recovered from georeferencing collector’s notes using point-radius method described in the Methods section. Abbreviations: UMMZ (University of Michigan Museum of Zoology, Ann Arbor, Michigan), MUSM (Museo de Historia Natural Universidad Nacional Mayor de San Marcos, Lima, Peru), FMNH (Field Museum of Natural History, Chicago, Illinois); for specimens without a museum catalog number assigned, the collector number (in lowercase) is provided. Population numbers are listed in square brackets; numbers of populations with genetic data are in bold.

ANCASH: *Huaraz*, Tullparaju, 4300, -9.03; -77.67 (FMNH 81371, 81373) [1]; *Huari*, Canrash, 4370 m, -9.68; -77.05 (mcp 54, 55, 56, 59, 63) [2]; *Huari*, Chacacmonte, 4220 m, -9.68; -77.11 (mcp 34, 35, 36, 42) [3]; *Huari*, Jupro, 4000 m, -9.59; -77.08 (mcp 67, 68) [4]; Huari, Paccha, 4240 m, -9.63; -77.12 (mcp 04, 05, 11, 15, 18, 20) [5]; *Huari*, Pumahuain, 4250 m, -9.66; -77.13 (mcp 24) [6]; *Huari*, Río Mosna, between Chavín and San Marcos, 3100 m, -9.55;-77.17 (FMNH 129213, 129215, 129219, 129225) [7]; *Santa*, Macate, 2250 m, -8.77; -78.08 (FMNH 20899, 20905, 20909) [8].

CAJAMARCA: *Cajamarca*, Cajamarca, 2890 m, -7.17; -78.51 (FMNH 19275, 19277, 19281, 19283) [9]; *Celendín*, Hacienda Limón, 2750 m, -6.83; -78.08 (FMNH 19285, 19287) [10]; *Contumaza*, Bosque Cachil, entre Cascas y Contumaza, 3050 m, -7.39; -78.78 (jaa 178, vpt 1674) [11]; *Cutervo*, San Andrés de Cutervo, 2070 m, -6.24; -78.72 (llw 1197, 1212) [12]; *Cutervo*, San Andrés de Cutervo, Cutervo National Park, 100 m over El Tragadero, 2910 m, -6.25; -78.77 (llw 1085, 1086, 1087, 1088, 1089, 1093, 1094, 1095, 1096, 1098, 1100, 1102, 1106, 1108, 1109, 1112, 1114, 1115, 1120, 1122, 1126, 1131, 1132, 1136, 1140, 1143, 1145, 1148, 1151, 1153, 1154, 1155, 1161, 1164, 1165, 1166, 1167, 1169, 1173, 1174, 1179, 1180, 1181, 1183) [13]; *Cutervo*, San Andrés de Cutervo, 4 km W San Andrés de Cutervo, 2250 m, -6.26; -78.72 (jaa 135, 139, jlm 175, vpt 1597) [14]; *San Ignacio*, Tabaconas, Cerro Coyona (Tabaconas-Namballe National Sanctuary), 3310 m, -5.23; -79.28 (jaa 805) [15]; *San Ignacio*, Tabaconas, Piedra Cueva in Cerro Coyona (Tabaconas-Namballe National Sanctuary), 3010 m, -5.27; -79.27 (llw 926, 929, 930, 946, 967, 976) [16]; *San Ignacio*, Tabaconas, Cerro Coyona (Tabaconas-Namballe National Sanctuary), 2790 m, -5.27; -79.27 (llw 995) [17]; *San Ignacio*, Tabaconas, Cerro La Viuda (Tabaconas-Namballe National Sanctuary's Buffer Zone), Campamento 1, 2760 m, -5.29; -79.34 (llw 1003, 1004, 1013, 1023) [18]; *San Ignacio*, Tabaconas, Cerro La Viuda (Tabaconas-Namballe National Sanctuary's Buffer Zone), Campamento 2, 2240 m, -5.28; -79.32 (llw 1048, 1082) [19]; *San Miguel*, La Florida, Agua Azul, 1660 m, -6.89; -79.08 (llw 491, 503, 511) [20]; *Santa Cruz*, 2.5 km E Monteseco, 2120 m, -6.85; -79.09 (jlm 208, vpt 1636) [21]; *Santa Cruz*, Catache, 3.81 km NE from Monteseco, 2150 m, -6.82; -79.08 (lhl 92, 93, 116, llw 1241, 1248, 1249, 1273, 1274) [22].

LA LIBERTAD: *Otuzco*, Hacienda Llagueda, 2250 m, -7.72; -78.72 (FMNH 19317, 19321, 19325) [23]; *Otuzco*, Summit between Otuzco and Llagueda, 2850 m, -7.90; -78.58 (FMNH 19849, 19851) [24]; *Sánchez Carrión*, Sanagorán, 2850 m, -7.79; -78.14 (lhl 85, llw 1219, 1220, 1221, 1222, 1225, 1226, 1227, 1232) [25]; *Sánchez Carrión*, Sanagorán, 2750 m, -7.78; -78.15 (vpt 2251, 2252, 2263) [26]; *Santiago de Chuco*, Cachicadán, 2870 m, -8.06; -78.17 (vpt 2277) [27]; *Santiago de Chuco*, Campamento Callacuyán, Quebrada Quishuara Sur, 4040 m, -7.94; -78.23 (avg 143) [28]; *Santiago de Chuco*, Campamento Callacuyán, arriba de Laguna Negra, 4020 m, -7.95; -78.24 (mvc 313, 323) [29]; *Santiago de Chuco*, Campamento Callacuyán, hondonada Laguna Viscachas, 4110 m, -7.95; -78.23 (mvc 325, 326) [30]; *Santiago de Chuco*, Campamento Callacuyán, Bosque de Polylepis, 3990 m, -7.92; -78.25 (mvc 333) [31]; *Santiago de Chuco*, Campamento Callacuyán, Laguna Pozo Hondo, 4130 m, -7.95; -78.25 (vpt 2380, 2387, 2390) [32]; *Trujillo*, Menocucho, 540 m, -8.02; -78.8 (FMNH 19329, 19333, 19335, 19343) [33].

PIURA: *Ayabaca*, Ayabaca, 2870 m, -4.63; -79.72 (FMNH 81379) [34]; *Huancabamba*, Canchaque, 1200, -5.40; -79.60 (FMNH 81357) [35]; *Huancabamba*, 2 km S of Canchaque, 1320, -5.50; -79.60 (FMNH 83441) [36]; *Huancabamba*, El Carmen de la Frontera, Carmen de la Frontera, Alto Samaniego, 2360 m, -5.11; -79.35 (ucf 43) [37]; *Huancabamba*, Huancabamba, 2020 m, -5.23; -79.47 (FMNH 84203) [38]; *Huancabamba*, Huancabamba, km 30 on road to San Ignacio, 2430 m, -5.25; -79.48 (FMNH 81353, 81363, 81365, 81367) [39]; *Huancabamba*, Tambo, 2870 m, -5.35; -79.55 (FMNH 81359) [40].

## Competing interests

The authors declare that they have no competing interests.

## Authors’ contributions

DFA participated in the conception and design of the study, carried out the ecomorphological and molecular analyses, and drafted the manuscript. LL compiled the samples, generated the morphometric and genetic data, and participated in the design of the study. LLK participated in the conception and design of the study and helped to draft the manuscript. All authors read and approved the final manuscript.

## Supplementary Material

Additional file 1 **Position of individuals of *A. mollis *in geographic, morphological, and environmental space.** Three-D scatterplot of populations according to their geographic coordinates and their individuals’ mean score on the first component of the Between Groups-PCA [46] on size-corrected shape data **(a)**. For comparison a similar three-D scatterplot of associated environmental variation among sampling localities, summarized by the first principal component of a PCA on all 21 environmental variables (Additional file 3: Table S3), is presented in **(b)**. The environmental variation is also represented by a scatterplot of the first and second principal component of this latter PCA (c). Note that a MANOVA on these PC scores showed that ecosystems are significantly different from each other in their environmental conditions (Wilks’ λ < 0.01, F5/34 = 8.56, p < 0.01). The position of puna individuals is indicated by the dashed ellipse. Symbols follow Figure 1. Click here for file

Additional file 2 **Matrix of pairwise genetic distances between *A. mollis *populations.** Genetic distances, estimated in Arlequin [114] as average number of pairwise nucleotide differences between populations based on a 1123 bp-region of the mitochondrial cytochrome b gene, is presented in **(a)** for populations with genetic data available (see Table 2). The dotted lines on perimeter of the plot frame point to the separation between populations according to the ecosystem they pertain to. The location of these populations with genetic data is shown in the ecosystem map in **(b)** (abbreviations as in Figure 1), with the genetically most different populations indicated by colored arrows corresponding to colors used in x and y axes in **(a)**; specific locations of the numbered populations can be found in the appendix. Click here for file

Additional file 3 Extended summary of environmental principal components and PLS axes. Click here for file

Additional file 4 Description of the location of the 54 landmarks (L.) used in the study. Click here for file

Additional file 5 Summary of allometry analyses. Click here for file

## References

[B1] Majerus MEN Mundy NI Mammalian melanism: natural selection in black and white Trends Genet 2003 19 585 588 10.1016/j.tig.2003.09.003 14585605

[B2] Luxbacher AM Knouft JH Assessing concurrent patterns of environmental niche and morphological evolution among species of horned lizards (*Phrynosoma*) J Evol Biol 2009 22 1669 1678 10.1111/j.1420-9101.2009.01779.x 19538346

[B3] Binning SA Chapman LJ Dumont J Feeding and breathing: trait correlations in an African cichlid fish J Zool 2010 282 140 149

[B4] Ashton KG Tracy MC de Queiroz A Is Bergmann's rule valid for mammals? Am Nat 2000 156 390 415 10.1086/303400 29592141

[B5] Eronen JT Puolamaki K Liu L Lintulaakso K Damuth J Janis C Fortelius M Precipitation and large herbivorous mammals I: estimates from present-day communities Evol Ecol Res 2010 12 217 233

[B6] Polly PD Eronen JT Fred M Dietl GP Mosbrugger V Scheidegger C Frank DC Damuth J Stenseth NC Fortelius M History matters: ecometrics and integrative climate change biology Proc R Soc Lond B Biol Sci 2011 278 1131 1140 10.1098/rspb.2010.2233 PMC3049084 21227966

[B7] Levins R Evolution in changing environments; some theoretical explorations 1968 Princeton: Princeton University Press

[B8] Donaldson-Matasci MC Lachmann M Bergstrom CT Phenotypic diversity as an adaptation to environmental uncertainty Evol Ecol Res 2008 10 493 515

[B9] Moran NA The evolutionary maintenance of alternative phenotypes Am Nat 1992 139 971 989 10.1086/285369

[B10] Terborgh J Distribution on environmental gradients - theory and preliminary interpretation of distributional patterns in avifauna of Cordillera Vilcabamba, Peru Ecology 1970 52 23 40

[B11] Patterson BD Stotz DF Solari S Fitzpatrick JW Pacheco V Contrasting patterns of elevational zonation for birds and mammals in the Andes of southeastern Peru J Biogeogr 1998 25 593 607 10.1046/j.1365-2699.1998.2530593.x

[B12] Lomolino MV Riddle BR Brown JH Biogeography 2006 Sunderland: Sinauer Associates

[B13] McCain CM Grytnes JA Elevational gradients in species richness Encyclopedia of Life Sciences 2010 Chichester: John Wiley & Sons, Ltd 1 10

[B14] Janzen DH Why mountain passes are higher in tropics Am Nat 1967 101 233 249 10.1086/282487

[B15] Ghalambor CK Huey RB Martin PR Tewksbury JJ Wang G Are mountain passes higher in the tropics? Janzen's hypothesis revisited Integr Comp Biol 2006 46 5 17 10.1093/icb/icj003 21672718

[B16] Knowles LL Alvarado-Serrano DF Exploring the population genetic consequences of the colonization process with spatio-temporally explicit models: insights from coupled ecological, demographic and genetic models in montane grasshoppers Mol Ecol 2010 19 3727 3745 10.1111/j.1365-294X.2010.04702.x 20723059

[B17] Svanbäck R Schluter D Niche specialization influences adaptive phenotypic plasticity in the threespine stickleback Am Nat 2012 180 50 59 10.1086/666000 22673650

[B18] Hallsson LR Björklund M Selection in a fluctuating environment leads to decreased genetic variation and facilitates the evolution of phenotypic plasticity J Evol Biol 2012 25 1275 1290 10.1111/j.1420-9101.2012.02512.x 22519748

[B19] Carleton MD Musser GG Wilson DE, Reeder DM Order Rodentia Mammal species of the world: a taxonomic and geographic reference 2005 Baltimore: John Hopkins University Press 894 1531

[B20] Diaz de Pascual A The rodent community of the Venezuelan cloud forest, Merida Pol Ecol Stud 1994 20 155 161

[B21] Barnett AA Small mammals of the Cajas Plateau, southern Ecuador: ecology and natural history Bull Florida Mus Nat Hist 1999 42 161 217

[B22] Dorst J Premières recherches sur la densité, la biomasse et la spécialisation écologique de quelques Rongeurs des hautes Andes du Pérou C R Seances Acad Sci D 1972 274 940 942 4622883

[B23] Alvarado-Serrano DF Caracterización morfométrica y distribución del género Akodon (Muridae: Sigmodontinae) en Ecuador 2005 Quito: B.Sc. Thesis. Pontificia Universidad Católica del Ecuador, Faculta de Ciencias Exactas y Naturales

[B24] Patton JL Smith MF Young KR, Valencia N Evolution and systematics of akodontine rodents (Muridae: Sigmodontinae) of Peru, with emphasis on the genus *Akodon* Memorias del Museo de Historia Natural. Volume 21 1992 Lima: Universidad Nacional Mayor de San Marcos 83 103

[B25] Grieco TM Rizk OT Cranial shape varies along an elevation gradient in Gambel's white-footed mouse (*Peromyscus maniculatus gambelii*) in the Grinnell resurvey Yosemite transect J Morphol 2010 271 897 909 10.1002/jmor.10839 20623653

[B26] Myers P Lundrigan BL Gillespie BW Zelditch ML Phenotypic plasticity in skull and dental morphology in the prairie deer mouse (*Peromyscus maniculatus bairdii*) J Morphol 1996 229 229 237 10.1002/(SICI)1097-4687(199608)229:2<229::AID-JMOR7>3.0.CO;2-W 8755340

[B27] Monteiro LR Duarte LC dos Reis SF Environmental correlates of geographical variation in skull and mandible shape of the punaré rat *Thrichomys apereoides* (Rodentia: Echimyidae) J Zool 2003 261 47 57 10.1017/S0952836903003893

[B28] Lalis A Baylac M Cosson JF Makundi RH Machang'u RS Denys C Cranial morphometric and fine scale genetic variability of two adjacent *Mastomys natalensis* (Rodentia: Muridae) populations Acta Theriol 2009 54 171 181 10.1007/BF03193173

[B29] Renaud S Michaux JR Adaptive latitudinal trends in the mandible shape of *Apodemus* wood mice J Biogeogr 2003 30 1617 1628 10.1046/j.1365-2699.2003.00932.x

[B30] Pergams ORW Lacy RC Rapid morphological and genetic change in Chicago-area *Peromyscus* Mol Ecol 2008 17 450 463 10.1111/j.1365-294X.2007.03517.x 17892465

[B31] Hautier L Lebrun R Cox PG Patterns of covariation in the masticatory apparatus of hystricognathous rodents: Implications for evolution and diversification J Morphol 2012 273 1319 1337 10.1002/jmor.20061 22833466

[B32] Williams SH Peiffer E Ford S Gape and bite force in the rodents *Onychomys leucogaster* and *Peromyscus maniculatus*: does jaw-muscle anatomy predict performance? J Morphol 2009 270 1338 1347 10.1002/jmor.10761 19480012

[B33] Cordero GA Epps CW From desert to rainforest: phenotypic variation in functionally important traits of bushy-tailed woodrats (*Neotoma cinerea*) across two climatic extremes J Mamm Evol 2012 19 135 153 10.1007/s10914-012-9187-0

[B34] Yom-Tov Y Yom-Tov S Climatic change and body size in two species of Japanese rodents Biol J Linn Soc 2004 82 263 267 10.1111/j.1095-8312.2004.00357.x

[B35] Terborgh J Bird species diversity on an Andean elevational gradient Ecology 1977 58 1007 1019 10.2307/1936921

[B36] Quintanilla V Comparación entre dos ecosistemas tropoandinos: la puna chilena y el páramo ecuatoriano Inf Geogr (Chile) 1983 30 25 45

[B37] Sklenár P Ramsay PM Diversity of zonal páramo plant communities in Ecuador Divers Distrib 2001 7 113 124 10.1046/j.1472-4642.2001.00101.x

[B38] Mena Vásconez P Hofstede R Moraes M, Ollgaard L, Kvist LP, Balslev H Los páramos ecuatorianos Botánica Económica de los Andes Centrales 2006 La Paz: Universidad Mayor de San Andrés 91 109

[B39] Simpson BB An historical phytogeography of the High Andean Flora Rev Chil Hist Nat 1983 56 109 122

[B40] Kuentz A de Mera AG Ledru MP Thouret JC Phytogeographical data and modern pollen rain of the puna belt in southern Peru (Nevado Coropuna, Western Cordillera) J Biogeogr 2007 34 1762 1776 10.1111/j.1365-2699.2007.01728.x

[B41] Cabrera AL Geo-ecología de las regiones montañosas de las américas tropicales Coll Geogr (Bonn) 1968 9 91 116

[B42] Tovar Serpa O Las gramíneas de Huancavelica Mem Mus Hist Natu Nac Mayor San Marcos 1957 6 1 110

[B43] Viscosi V Cardini A Leaf morphology, taxonomy and geometric morphometrics: a simplified protocol for beginners PLoS One 2011 6 e25630 10.1371/journal.pone.0025630 21991324 PMC3184990

[B44] Klingenberg CP Barluenga M Meyer A Shape analysis of symmetric structures: quantifying variation among individuals and asymmetry Evolution 2002 56 1909 1920 12449478 10.1111/j.0014-3820.2002.tb00117.x

[B45] Olson DM Dinerstein E Wikramanayake ED Burgess ND Powell GVN Underwood EC D'Amico JA Itoua I Strand HE Morrison JC Terrestrial ecoregions of the worlds: a new map of life on earth Bioscience 2001 51 933 938 10.1641/0006-3568(2001)051[0933:TEOTWA]2.0.CO;2

[B46] Boulesteix AL A note on between-group PCA Int J Pure Appl Math 2005 19 359 366

[B47] Bookstein FL Sampson PD Streissguth AP Barr HM Measuring "dose" and "response" with multivariate data using partial least squares techniques Commun Stat- A Theor 1990 19 765 804 10.1080/03610929008830231

[B48] Zelditch ML Swiderski DL Sheets HD Fink WL Geometric morphometrics for biologists: a primer 2004 Amsterdam: Elsevier Academic Press

[B49] Griffith DA Peres-Neto PR Spatial modeling in ecology: the flexibility of eigenfunction spatial analyses Ecology 2006 87 2603 2613 10.1890/0012-9658(2006)87[2603:SMIETF]2.0.CO;2 17089668

[B50] Perez SI Diniz JAF Bernal V Gonzalez PN Spatial regression techniques for inter-population data: studying the relationships between morphological and environmental variation J Evol Biol 2010 23 237 248 10.1111/j.1420-9101.2009.01905.x 20002248

[B51] Fadda C Corti M Three-dimensional geometric morphometrics of *Arvicanthis*: implications for systematics and taxonomy J Zoolog Syst Evol Res 2001 39 235 245 10.1046/j.1439-0469.2001.00169.x

[B52] Caumul R Polly PD Phylogenetic and environmental components of morphological variation: skull, mandible, and molar shape in marmots (*Marmota*, Rodentia) Evolution 2005 59 2460 2472 16396186

[B53] Cardini A Jansson AU Elton S A geometric morphometric approach to the study of ecogeographical and clinal variation in vervet monkeys J Biogeogr 2007 34 1663 1678 10.1111/j.1365-2699.2007.01731.x

[B54] Márchan-Rivadeneira MR Larsen PA Phillips CJ Strauss RE Baker RJ On the association between environmental gradients and skull size variation in the great fruit-eating bat, *Artibeus lituratus* (Chiroptera: Phyllostomidae) Biol J Linn Soc 2012 105 623 634 10.1111/j.1095-8312.2011.01804.x

[B55] Samuels JX Cranial morphology and dietary habits of rodents Zool J Linn Soc 2009 156 864 888 10.1111/j.1096-3642.2009.00502.x

[B56] Wolf M Friggens M Salazar-Bravo J Does weather shape rodents? Climate related changes in morphology of two heteromyid species Naturwissenschaften 2009 96 93 101 10.1007/s00114-008-0456-y 18843477

[B57] Voss RS A new species of *Thomasomys* (Rodentia: Muridae) from eastern Ecuador, with remarks on mammalian diversity and biogeography in the Cordillera Oriental Am Mus Novit 2003 3421 1 47

[B58] Smith MF Patton JL Kelt DA, Lessa EP, Salazar-Bravo J, Patton JL Molecular phylogenetics and diversification of South American grass mice, genus *Akodon* The Quintessential Naturalist: Honoring the Life and Legacy of Oliver P Pearson. Volume 134 2007 Berkeley: University of California Publications: Zoology 827 858

[B59] Losos JB Ecomorphology, performance capability, and scaling of West-Indian *Anolis* lizards: an evolutionary analysis Ecol Monogr 1990 60 369 388 10.2307/1943062

[B60] Lahti DC Johnson NA Ajie BC Otto SP Hendry AP Blumstein DT Coss RG Donohue K Foster SA Relaxed selection in the wild Trends Ecol Evol 2009 24 487 496 10.1016/j.tree.2009.03.010 19500875

[B61] Tinker MT Guimaraes PR Novak M Marquitti FMD Bodkin JL Staedler M Bentall G Estes JA Structure and mechanism of diet specialisation: testing models of individual variation in resource use with sea otters Ecol Lett 2012 15 475 483 10.1111/j.1461-0248.2012.01760.x 22414160

[B62] Van Valen L Morphological variation and width of ecological niche Am Nat 1965 99 377 390 10.1086/282379

[B63] Price T Diet variation in a population of Darwin's finches Ecology 1987 68 1015 1028 10.2307/1938373

[B64] Bolnick DI Svanback R Fordyce JA Yang LH Davis JM Hulsey CD Forister ML The ecology of individuals: Incidence and implications of individual specialization Am Nat 2003 161 1 28 10.1086/343878 12650459

[B65] Smartt RA Lemen CA Intrapopulational morphological variation as a predictor of feeding behavior in deer mice Am Nat 1980 116 891 894 10.1086/283679

[B66] Pearson OP Ralph CP The diversity and abundance of vertebrates along an altitudinal gradient in Peru Mem Mus Hist Natu U Nac Mayor San Marcos 1978 18 1 97

[B67] Mani MS Ecology and biogeography of high altitude insects 1968 The Hague: Dr. W. Junk N. V. Publishers

[B68] Sarmiento G Vuilleumier F, Monasterio M Ecological features of climate in high tropical mountains High altitude tropical biogeography 1986 Oxford: Oxford University Press 11 45

[B69] Hammond KA Szewczak J Krol E Effects of altitude and temperature on organ phenotypic plasticity along an altitudinal gradient J Exp Biol 2001 204 1991 2000 11441040 10.1242/jeb.204.11.1991

[B70] Santana S Grosse IR Dumont ER Dietary hardness, loading behavior, and the evolution of skull form in bats Evolution 2012 66 2587 2598 10.1111/j.1558-5646.2012.01615.x 22834755

[B71] Lieberman DE Krovitz GE Yates FW Devlin M Claire MS Effects of food processing on masticatory craniofacial growth in a retrognathic strain and face J Hum Evol 2004 46 655 677 10.1016/j.jhevol.2004.03.005 15183669

[B72] Pizzimenti JJ Desalle R Dietary and morphometric variation in some Peruvian rodent communities: the effect of feeding strategy on evoltuion Biol J Linn Soc Lond 1980 13 263 285 10.1111/j.1095-8312.1980.tb00087.x

[B73] Hammond KA Roth J Janes DN Dohm MR Morphological and physiological responses to altitude in deer mice *Peromyscus maniculatus* Physiol Biochem Zool 1999 72 613 622 10.1086/316697 10521329

[B74] Rosenblum EB Harmon LJ “Same same but different": replicated ecological speciation at white sands Evolution 2011 65 946 960 10.1111/j.1558-5646.2010.01190.x 21073450

[B75] Hoekstra HE Krenz JG Nachman MW Local adaptation in the rock pocket mouse (*Chaetodipus intermedius*): natural selection and phylogenetic history of populations Heredity 2005 94 217 228 10.1038/sj.hdy.6800600 15523507

[B76] Steiner CC Weber JN Hoekstra HE Adaptive variation in beach mice produced by two interacting pigmentation genes PLoS Biol 2007 5 1880 1889 10.1371/journal.pbio.0050219 PMC1945039 17696646

[B77] Slatkin M Gene flow in natural populations Ann Rev Ecol Syst 1985 16 393 430

[B78] Smith JA Seltzer GO Rodbell DT Klein AG Regional synthesis of last glacial maximum snowlines in the tropical Andes, South America Quatern Int 2005 138 145 167

[B79] Graham CH Ron SR Santos JC Schneider CJ Moritz C Integrating phylogenetics and environmental niche models to explore speciation mechanisms in dendrobatid frogs Evolution 2004 58 1781 1793 15446430 10.1111/j.0014-3820.2004.tb00461.x

[B80] Brumfield RT Edwards SV Evolution into and out of the Andes: a Bayesian analysis of historical diversification in *Thamnophilus* antshrikes Evolution 2007 61 346 367 10.1111/j.1558-5646.2007.00039.x 17348945

[B81] Sedano RE Burns KJ Are the Northern Andes a species pump for neotropical birds? Phylogenetics and biogeography of a clade of neotropical tanagers (Aves: Thraupini) J Biogeogr 2010 37 325 343 10.1111/j.1365-2699.2009.02200.x

[B82] Williams JW Jackson ST Novel climates, no-analog communities, and ecological surprises Front Ecol Environ 2007 5 475 482 10.1890/070037

[B83] Deutsch CA Tewksbury JJ Huey RB Sheldon KS Ghalambor CK Haak DC Martin PR Impacts of climate warming on terrestrial ectotherms across latitude Proc Natl Acad Sci USA 2008 105 6668 6672 10.1073/pnas.0709472105 18458348 PMC2373333

[B84] Sikes RS Gannon WL Amer Soc M Guidelines of the American Society of Mammalogists for the use of wild mammals in research J Mammal 2011 92 235 253 10.1644/10-MAMM-F-355.1 PMC5909806 29692469

[B85] D'Elía G Phylogenetics of sigmodontinae (Rodentia, Muroidea, Cricetidae), with special reference to the akodont group, and with additional comments on historical biogeography Cladistics 2003 19 307 323

[B86] Jayat JP Ortiz PE Salazar-Bravo J Pardiñas UFJ D'Elia G The *Akodon boliviensis* species group (Rodentia: Cricetidae: Sigmodontinae) in Argentina: species limits and distribution, with the description of a new entity Zootaxa 2010 2409 1 61

[B87] Hershkovitz P Notes on the distribution of the akodont rodent, *Akodon mollis*, in Ecuador with a description of a new race Occas Pap Mus Zool Univ Mich 1940 418 1 3

[B88] Myers P Patton JL *Akodon* of Peru and Bolivia-Revision of the *fumeus* group (Rodentia: Sigmodontinae) Misc Publ Mus Zool Univ Mich 1989 721 1 35

[B89] de Oliveira JA Strauss RE dos Reis SF Assessing relative age and age structure in natural populations of *Bolomys lasiurus* (Rodentia: Sigmodontinae) in northeastern Brazil J Mammal 1998 79 1170 1183 10.2307/1383008

[B90] Myers P Redford KH, Eisenberg JF A preliminary revision of the *varius* group of *Akodon* (*A*. *dayi*, *dolores*, *molinae*, *neocenus*, *simulator*, *toba*, and *varius*) Advances in Neotropical Mammalogy 1989 Gainsville: Sandhill Crane Press 5 54

[B91] Wieczorek J Guo QG Hijmans RJ The point-radius method for georeferencing locality descriptions and calculating associated uncertainty Int J Geogr Inf Sci 2004 18 745 767 10.1080/13658810412331280211

[B92] Farr TG Rosen PA Caro E Crippen R Duren R Hensley S Kobrick M Paller M Rodriguez E Roth L The shuttle radar topography mission Rev Geophys 2007 45 1 33

[B93] Graham CH Silva N Velasquez-Tibata J Evaluating the potential causes of range limits of birds of the Colombian Andes J Biogeogra 2010 37 1863 1875

[B94] McCormack JE Zellmer AJ Knowles LL Does niche divergence accompany allopatric divergence in *Aphelcoma* jays as predicted under ecological speciation? insights from tests with niche models Evolution 2010 64 1231 1244 19922442 10.1111/j.1558-5646.2009.00900.x

[B95] Hijmans RJ Cameron SE Parra JL Jones PG Jarvis A Very high resolution interpolated climate surfaces for global land areas Int J Climatol 2005 25 1965 1978 10.1002/joc.1276

[B96] Friedl MA McIver DK Hodges JCF Zhang XY Muchoney D Strahler AH Woodcock CE Gopal S Schneider A Cooper A Global land cover mapping from MODIS: algorithms and early results Remote Sens Environ 2002 83 287 302 10.1016/S0034-4257(02)00078-0

[B97] Imhoff ML Bounoua L Ricketts T Loucks C Harriss R Lawrence WT Global patterns in human consumption of net primary production Nature 2004 429 870 873 10.1038/nature02619 15215863

[B98] Environmental Systems Research Institute, Inc ArcGIS Desktop: Release 9.3 2009 Redlands

[B99] Gene Codes Corporation Sequencher v4.2 sequence analysis software 2006 Ann Arbor http://www.genecodes.com

[B100] Maddison WP Maddison DR MacClade: analysis of phylogeny and character evolution 1992 Sunderland: Sinauer Associates 10.1159/000156416 2606395

[B101] Cavallisforza LL Menozzi P Piazza A Demic expansions and human evolution Science 1993 259 639 646 10.1126/science.8430313 8430313

[B102] Wright S Isolation by distance. Genetics 1943 28 114 138 10.1093/genetics/28.2.114 PMC1209196 17247074

[B103] Mantel N The detection of disease clustering and a generalized regression approach Cancer Res 1967 27 209 220 6018555

[B104] Rohlf FJ tpsDIG2 v2.16 2010 Stony Brook: Department of Ecology and Evolution, State University of New York at Stony Brook [http://life.bio.sunysb.edu/morph/]

[B105] Dryden IL Mardia KV Statistical shape analysis 1998 Chichester: John Wiley & Sons

[B106] Klingenberg CP MorphoJ: an integrated software package for geometric morphometrics Mol Ecol Resour 2011 11 353 357 10.1111/j.1755-0998.2010.02924.x 21429143

[B107] Rohlf FJ Slice D Extensions of the procrustes method for the optimal superimposition of landmarks Syst Zool 1990 39 40 59 10.2307/2992207

[B108] R Core Development Team R: a language and environment for statistical computing v2.15.1 2012 Vienna [http://www.r-project.org]

[B109] Rohlf FJ tpsRegr v2.16 2011 Stony Brook: Department of Ecology and Evolution, State University of New York at Stony Brook http://life.bio.sunysb.edu/morph/

[B110] Seetah TK Cardini A Miracle PT Can morphospace shed light on cave bear spatial-temporal variation? Population dynamics of *Ursus spelaeus* from Romualdova pecina and Vindija, (Croatia) J Archaeol Sci 2012 39 500 510 10.1016/j.jas.2011.10.005

[B111] Escoufier Y Le traitement des variables vectorielles Biometrics 1973 29 751 760 10.2307/2529140

[B112] Abdi H Salkind N, Rasmussen K RV coefficient and congruence coefficient Encyclopedia of measurement and statistics 2007 Thousand Oaks: Sage Publications

[B113] Rangel TF Diniz JAF Bini LM SAM: a comprehensive application for Spatial Analysis in Macroecology Ecography 2010 33 46 50 10.1111/j.1600-0587.2009.06299.x

[B114] Excoffier L Laval G Schneider S Arlequin version 3.0: an integrated software package for population genetics data analysis Evol Bioinform Online 2005 1 47 50 19325852 PMC2658868

