# Supplementary material for: Localized versus generalist phenotypes in a broadly distributed tropical mammal: how is intraspecific variation distributed across disparate environments?
Source: BMC Evol Biol. 2013 Jul 31;13:160. doi: 10.1186/1471-2148-13-160 (PMC3737017; doi:10.1186/1471-2148-13-160)
Supplement: Additional file 1 — Position of individuals of A. mollis in geographic, morphological, and environmental space. Three-D scatterplot of populations according to their geographic coordinates and their individuals’ mean score on the first component of the Between Groups-PCA [46] on size-corrected shape data (a). For comparison a similar three-D scatterplot of associated environmental variation among sampling localities, summarized by the first principal component of a PCA on all 21 environmental variables (Additional file 3: Table S3), is presented in (b). The environmental variation is also represented by a scatterplot of the first and second principal component of this latter PCA (c). Note that a MANOVA on these PC scores showed that ecosystems are significantly different from each other in their environmental conditions (Wilks’ λ < 0.01, F5/34 = 8.56, p < 0.01). The position of puna individuals is indicated by the dashed ellipse. Symbols follow Figure 1. [file 1471-2148-13-160-S1.pdf]

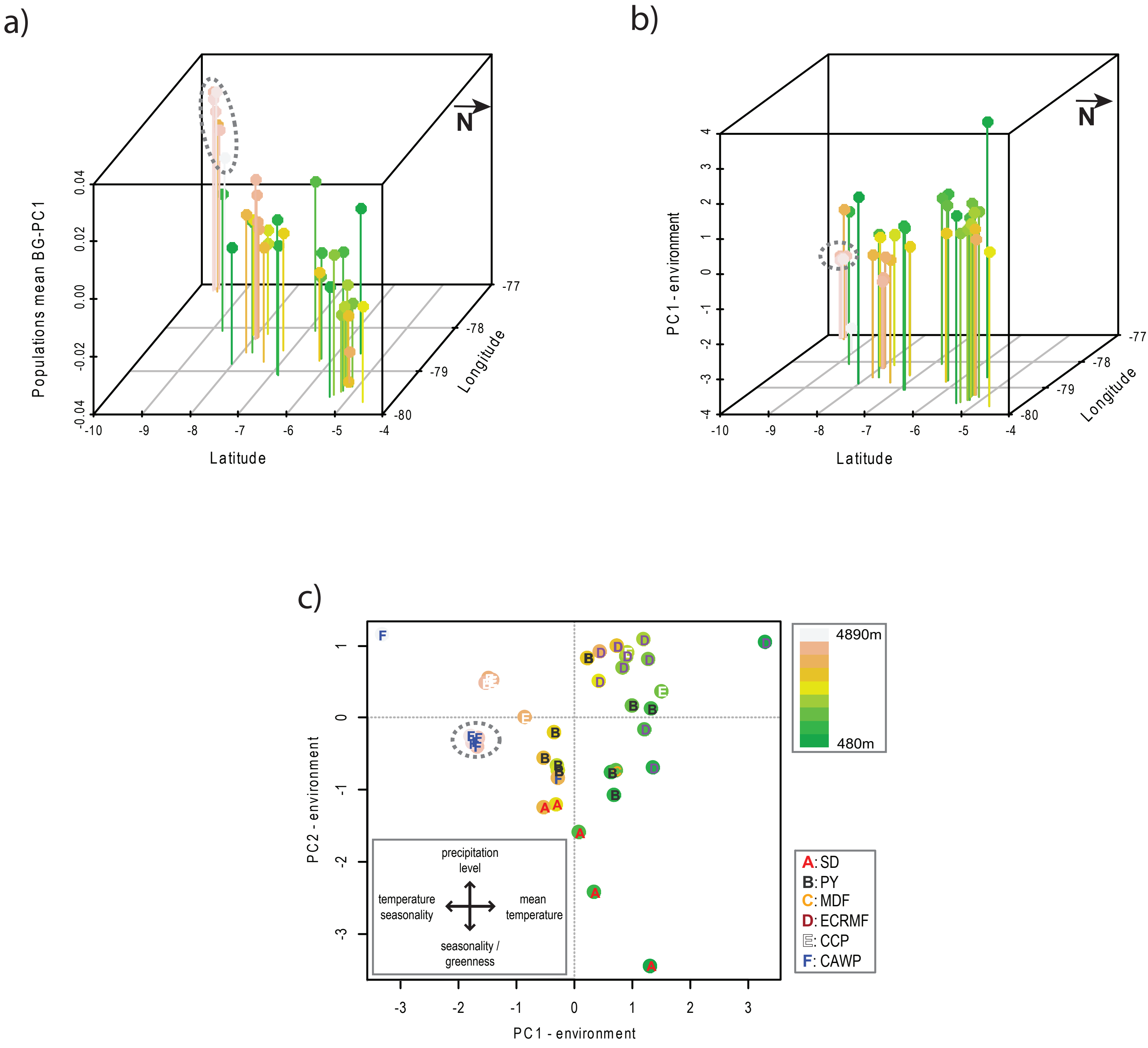


**Supplementary Figure 1 – Position of individuals of *A. mollis* in geographic, morphological, and environmental space**

Three-D scatterplot of populations according to their geographic coordinates and their individuals' mean score on the first component of the Between Groups-PCA [38] on size-corrected shape data (a). For comparison a similar three-D scatterplot of associated environmental variation among sampling localities, summarized by the first principal component of a PCA on all 21 environmental variables (Table S3), is presented in (b). The environmental variation is also represented by a scatterplot of the first and second principal component of this latter PCA (c). Note that a MANOVA on these PC scores showed that ecosystems are significantly different from each other in their environmental conditions (Wilks' $\lambda < 0.01$, $F_{5/34} = 8.56$, $p < 0.01$). The position of puna individuals is indicated by the dashed ellipse. Symbols follow Fig. 1.
