# Supplementary material for: Localized versus generalist phenotypes in a broadly distributed tropical mammal: how is intraspecific variation distributed across disparate environments?
Source: BMC Evol Biol. 2013 Jul 31;13:160. doi: 10.1186/1471-2148-13-160 (PMC3737017; doi:10.1186/1471-2148-13-160)
Supplement: Additional file 2 — Matrix of pairwise genetic distances between A. mollis populations. Genetic distances, estimated in Arlequin [114] as average number of pairwise nucleotide differences between populations based on a 1123 bp-region of the mitochondrial cytochrome b gene, is presented in (a) for populations with genetic data available (see Table 2). The dotted lines on perimeter of the plot frame point to the separation between populations according to the ecosystem they pertain to. The location of these populations with genetic data is shown in the ecosystem map in (b) (abbreviations as in Figure 1), with the genetically most different populations indicated by colored arrows corresponding to colors used in x and y axes in (a); specific locations of the numbered populations can be found in the appendix. [file 1471-2148-13-160-S2.pdf]

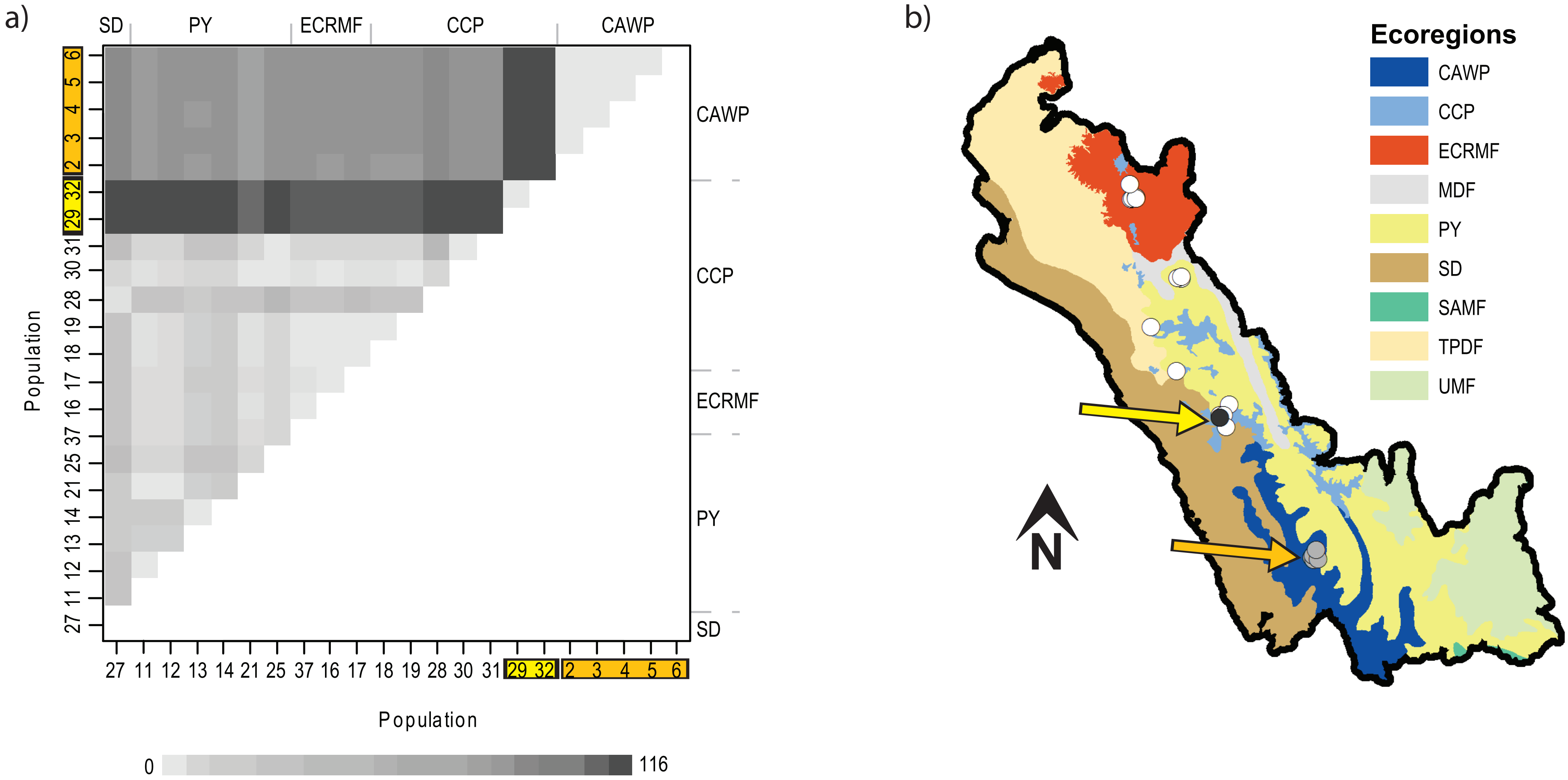

Supplementary Figure 2 – Matrix of pairwise genetic distances between *A. mollis* populations

Genetic distances, estimated in Arlequin [114] as average number of pairwise nucleotide differences between populations based on a 1123bp-region of the mitochondrial cytochrome b gene, is presented in (a) for populations with genetic data available (see Table 2). The dotted lines on perimeter of the plot frame point to the separation between populations according to the ecosystem they pertain to. The location of these populations with genetic data is shown in the ecosystem map in (b) (abbreviations as in Fig. 1), with the genetically most different populations indicated by colored arrows corresponding to colors used in x and y axes in (a); specific locations of the numbered populations can be found in the appendix.
