# Supplementary material for: Localized versus generalist phenotypes in a broadly distributed tropical mammal: how is intraspecific variation distributed across disparate environments?
Source: BMC Evol Biol. 2013 Jul 31;13:160. doi: 10.1186/1471-2148-13-160 (PMC3737017; doi:10.1186/1471-2148-13-160)
Supplement: Additional file 3 — Extended summary of environmental principal components and PLS axes. [file 1471-2148-13-160-S3.doc]

### Supplementary Table 1 – Description of the location of the 54 landmarks (L.) used in the study.

| L. | Description |
| --- | --- |
| 1 | most anterior border of the gnathic process |
| 2,3 | outermost curvature of the alveolus of the incisor |
| 4,5 | most anterior border of the incisor foramen |
| 6,7 | outermost suture between the premaxilla and maxilla |
| 8 | point of convergence of the ventral nasal concha |
| 9,10 | base of the zygomatic plate at the level of the masseteric tubercle |
| 11,12 | most posterior border of the incisor foramen |
| 13, 14 | paracone of first upper molar |
| 15,16 | paracone of second upper molar |
| 17,18 | most posterior border of third upper molar |
| 19, 20 | center of the posterior palatine foramen |
| 21 | medial anterior border of the mesopterygoid fossa |
| 22 | medial suture between the presphenoid and basisphenoid |
| 23, 24 | outermost suture between presphenoid and basisphenoid |
| 25, 26 | suture of the alisphenoid and the frontal at the level of the squamosal-alisphenoid groove |
| 27, 28 | groove at suture between the frontal and squamosal |
| 29, 30 | most anterior curvature of the squamosal projection that creates the orbit |
| 31, 32 | suture of the alisphenoid and squamosal at the level of the squamosal-alisphenoid groove |
| 33, 34 | most anterior border of the foramen ovale |
| 35, 36 | center of the posterior curvature of the Eustachian tube |
| 37, 38 | most anterior border of the tympanic thickening of the bulla |
| 39, 40 | anterior border of the carotid canal; L41: Most anterior medial margin of the basioccipital |
| 42, 43 | most anterior external border of the ectotympanic |
| 44,45 | most posterior border of the masseteric tubercle |
| 46, 47 | innermost opening of the auditory meatus |
| 48, 49 | opening of the basioccipital at the level of the occipital condyle |
| 50 | most anterior medial border of the foramen magnum |
| 51, 52 | most anterior border of the paramastoid process |
| 53, 54 | most external border of the occipital condyle |
