# Supplementary material for: Localized versus generalist phenotypes in a broadly distributed tropical mammal: how is intraspecific variation distributed across disparate environments?
Source: BMC Evol Biol. 2013 Jul 31;13:160. doi: 10.1186/1471-2148-13-160 (PMC3737017; doi:10.1186/1471-2148-13-160)
Supplement: Additional file 4 — Description of the location of the 54 landmarks (L.) used in the study. [file 1471-2148-13-160-S4.doc]

### Supplementary Table 2 – Summary of allometry analyses.

Summary of regressions of Procrustes projected landmarks onto log_e_-transformed centroid size run independently for each ecosystem (abbreviations as in Fig. 1). Significance was assessed with 10000 permutations.

| Group | % predicted | p-value |
| --- | --- | --- |
| CAWP | 7.79 | 0.025 |
| CCP | 9.38 | 0.207 |
| ECRMF | 6.65 | 0.287 |
| MDF | - | - |
| PY | 4.22 | <0.001 |
| SD | 11.24 | 0.162 |
