# Supplementary material for: Localized versus generalist phenotypes in a broadly distributed tropical mammal: how is intraspecific variation distributed across disparate environments?
Source: BMC Evol Biol. 2013 Jul 31;13:160. doi: 10.1186/1471-2148-13-160 (PMC3737017; doi:10.1186/1471-2148-13-160)
Supplement: Additional file 5 — Summary of allometry analyses. [file 1471-2148-13-160-S5.doc]

## Supplementary Table 3 – Extended summary of environmental principal components and PLS axes.

Principal component loadings of 21 standardized environmental variables used in the ecomorphological analyses for all PCs with an eigenvalue bigger than 1. In addition, the loading of these 10 PCs in the PLS axes, as well as the correlation between these PCs and elevation is provided in the bottom rows. For the correlation with elevation, Pearson’s r coefficient is shown in the first line, the associated p-value, in the second line. Coefficients that remain significant after a Bonferroni correction are in bold).

| Variable | PC1 | PC2 | PC3 | PC4 | PC5 | PC6 | PC7 | PC8 | PC9 | PC10 |
| --- | --- | --- | --- | --- | --- | --- | --- | --- | --- | --- |
| Annual Mean Temperature | 0.93 | -0.29 | 0.19 | -0.01 | 0.06 | 0.00 | 0.04 | 0.01 | -0.02 | 0.00 |
| Mean Diurnal Temperature Range | -0.65 | -0.04 | 0.54 | 0.45 | 0.19 | -0.16 | 0.04 | 0.11 | 0.00 | 0.02 |
| Isothermality | 0.50 | 0.68 | 0.35 | 0.10 | 0.05 | -0.24 | -0.29 | 0.09 | 0.07 | -0.05 |
| Temperature Seasonality | -0.34 | -0.82 | -0.20 | -0.19 | 0.09 | 0.26 | 0.04 | 0.23 | 0.06 | -0.06 |
| Maximum Temperature of Warmest Month | 0.89 | -0.37 | 0.23 | 0.02 | 0.06 | 0.00 | 0.09 | 0.00 | -0.02 | 0.01 |
| Minimum Temperature of Warmest Month | 0.98 | -0.17 | 0.09 | -0.09 | 0.02 | 0.02 | 0.01 | -0.01 | -0.01 | -0.01 |
| Temperature Annual Range | -0.80 | -0.36 | 0.28 | 0.30 | 0.09 | -0.05 | 0.19 | 0.04 | -0.04 | 0.06 |
| Mean Temperature of Wettest Quarter | 0.90 | -0.38 | 0.17 | -0.04 | 0.06 | 0.03 | 0.03 | 0.03 | -0.01 | 0.00 |
| Mean Temperature of Driest Quarter | 0.95 | -0.23 | 0.20 | 0.00 | 0.04 | -0.01 | 0.04 | -0.01 | -0.02 | 0.00 |
| Mean Temperature of Warmest Quarter | 0.91 | -0.37 | 0.17 | -0.03 | 0.07 | 0.03 | 0.04 | 0.03 | -0.01 | 0.00 |
| Mean Temperature of Coldest Quarter | 0.95 | -0.23 | 0.20 | 0.00 | 0.05 | -0.02 | 0.04 | -0.01 | -0.02 | 0.00 |
| Annual Precipitation | -0.01 | 0.97 | 0.11 | 0.06 | -0.07 | 0.13 | 0.16 | 0.01 | 0.01 | -0.06 |
| Precipitation of Wettest Month | -0.22 | 0.66 | 0.62 | -0.33 | -0.01 | 0.06 | 0.09 | -0.01 | 0.09 | -0.01 |
| Precipitation of Driest Month | 0.52 | 0.80 | -0.25 | 0.10 | -0.10 | 0.03 | -0.01 | 0.06 | 0.01 | 0.06 |
| Precipitation Seasonality | -0.20 | -0.88 | 0.25 | -0.28 | 0.02 | 0.08 | -0.15 | -0.01 | 0.09 | 0.13 |
| Precipitation of Wettest Quarter | -0.22 | 0.76 | 0.52 | -0.24 | -0.14 | 0.05 | 0.11 | -0.05 | 0.05 | 0.01 |
| Precipitation of Driest Quarter | 0.52 | 0.81 | -0.21 | 0.07 | -0.11 | 0.01 | 0.02 | 0.09 | 0.00 | 0.06 |
| Precipitation of Warmest Quarter | -0.42 | 0.69 | 0.35 | -0.30 | 0.16 | 0.20 | -0.17 | 0.05 | -0.19 | 0.01 |
| Precipitation of Coldest Quarter | 0.53 | 0.80 | -0.20 | 0.07 | -0.12 | 0.04 | 0.05 | 0.11 | 0.00 | 0.07 |
| Net Primary Productivity | 0.26 | 0.67 | -0.15 | 0.36 | 0.48 | 0.29 | -0.04 | -0.10 | 0.08 | 0.01 |
| Normalized Difference Vegetation Index | 0.07 | -0.51 | 0.41 | 0.53 | -0.42 | 0.30 | -0.13 | -0.02 | -0.01 | -0.02 |
| Eigenvalue | 8.72 | 7.73 | 1.96 | 1.12 | 0.57 | 0.39 | 0.26 | 0.12 | 0.07 | 0.04 |
| Percentage variance explained | 41.52 | 36.83 | 9.32 | 5.32 | 2.72 | 1.87 | 1.22 | 0.56 | 0.35 | 0.21 |
| Scores on PLS1 – Size | -0.61 | -0.16 | -0.39 | -0.20 | 0.12 | -0.10 | 0.07 | -0.47 | -0.23 | 0.33 |
| Scores on PLS1 – Uncorrected Shape | 0.27 | 0.37 | -0.44 | -0.12 | -0.26 | -0.63 | -0.25 | -0.17 | 0.08 | 0.07 |
| Scores on PLS2 – Uncorrected Shape | -0.68 | 0.16 | -0.36 | 0.05 | -0.10 | -0.08 | 0.40 | 0.23 | 0.35 | 0.16 |
| Scores on PLS1 – Size-corrected Shape | 0.58 | 0.38 | -0.22 | -0.03 | -0.27 | -0.53 | -0.31 | 0.01 | 0.10 | -0.08 |
| Scores on PLS2 – Size-corrected Shape | -0.58 | 0.22 | -0.52 | -0.09 | -0.21 | -0.27 | 0.24 | -0.09 | 0.33 | 0.23 |
| Correlation with elevation | -0.87  (**<0.001**) | 0.42  (**<0.001**) | -0.03  (0.729) | 0.09  (0.248) | -0.11  (0.178) | -0.13  (0.099) | -0.01  (0.909) | -0.08  (0.350) | -0.05  (0.516) | 0.04  (0.648) |
